# Hierarchical Multimodal Fusion of Multi-Sequence MRI and Clinical Metadata for the Classification of Rotator Cuff Tears

**DOI:** 10.3390/jcm15145525

**Published:** 2026-07-14

**Authors:** Sergen Aşık, Ahmet Yazıcı, Murat Aşçı, İrfan Okumuşer

**Affiliations:** 1 Department of Software Engineering, Faculty of Engineering and Architecture, Eskişehir Osmangazi University, Eskişehir 26040, Türkiye; 2 Center of Intelligent Systems Applications Research, Eskişehir Osmangazi University, Eskişehir 26040, Türkiye; ayazici@ogu.edu.tr; 3 Department of Computer Engineering, Faculty of Engineering and Architecture, Eskişehir Osmangazi University, Eskişehir 26040, Türkiye; 4 Department of Orthopedics and Traumatology, Faculty of Medicine, Bilecik Şeyh Edebali University, Bilecik 11230, Türkiye; muratasci55@gmail.com; 5 Department of Radiology, Yunus Emre State Hospital, Ministry of Health, Eskişehir 26190, Türkiye; irfanokumuser@hotmail.com

**Keywords:** rotator cuff tear, shoulder MRI, multi-sequence MRI, deep learning, convolutional neural network, vision transformer, multimodal fusion, clinical metadata, sequence selection

## Abstract

**Background/Objectives**: Rotator cuff tears are a leading cause of shoulder disability. While multi-sequence MRI is standard, the optimal deep learning integration of heterogeneous image series and clinical metadata remains unresolved. This study evaluated a hierarchical, sequence-aware multimodal framework for patient-level binary rotator cuff tear classification. **Methods**: A single-center cohort of 199 patients (100 tears, 99 controls) was analyzed across four MRI sequences (T1 coronal, T2 fat-suppressed sagittal, and proton density [PD] fat-suppressed coronal and transverse/axial) and nine demographic features. Under a patient-level stratified three-fold cross-validation scheme preventing data leakage, we evaluated ResNet50 and Vision Transformer baselines (Study 0), full-protocol fusion topologies (Study 1), and systematically mapped sequence-subset combinations with or without metadata (Study 2). **Results**: In Study 0, the PD coronal ResNet50 model was the top baseline (AUC = 0.9834, F1 = 0.9515). In Study 1, late decision fusion yielded the highest AUC (0.9909), while feature concatenation optimized threshold balance (F1 = 0.9502). In Study 2, a streamlined three-sequence subset with metadata (C14M: T2 + PDc + PDt) achieved peak performance (AUC = 0.9961, 95% CI: 0.9823–0.9987, F1 = 0.9618, MCC = 0.9238), outperforming the full protocol (AUC = 0.9909, F1 = 0.9355). Metadata utility was configuration-dependent, assisting only fluid-sensitive combinations. **Conclusions**: Rather than indiscriminately aggregating entire clinical protocols, multimodal fusion is optimized by selecting complementary imaging series. For binary classification, excluding non-fat-suppressed T1 images in favor of a streamlined T2 and PD set stabilized by clinical demographics maximized classification performance in this internally validated, single-center cohort.

## 1. Introduction

Rotator cuff tears represent one of the most common and clinically consequential disorders of the shoulder, particularly in middle-aged and older adults. Their importance is not limited to the mere presence or absence of tendon discontinuity; tear size, tear chronicity, tendon retraction, muscle atrophy, fatty infiltration, and associated shoulder pathology all influence treatment decisions, surgical repairability, postoperative healing, and long-term function [1,2]. MRI is routinely used because it offers multiplanar soft-tissue contrast and can depict both direct tendon abnormalities and secondary signs, such as fluid-sensitive signal changes, muscle quality, bursal involvement, and adjacent joint findings [3,4,5,6]. Nevertheless, routine shoulder MRI interpretation remains demanding. Partial-thickness tears, small full-thickness tears, degenerative tendon changes, calcific tendinosis, subscapularis involvement, and postoperative or chronic changes can create diagnostic uncertainty even when high-quality imaging is available.

The increasing clinical and technical interest in artificial intelligence (AI) for shoulder MRI reflects this diagnostic complexity. Early and contemporary deep learning studies have demonstrated that convolutional neural networks, 3D networks, radiomics models, transformer-style comparators, multitask systems, and segmentation pipelines can detect or grade rotator cuff and supraspinatus-related abnormalities on MRI [7,8,9,10,11,12,13,14,15,16]. Recent systematic reviews of MRI-based rotator cuff tear AI models and broader shoulder AI applications further support the feasibility of automated interpretation, while also emphasizing limitations related to cohort size, external validation, scanner heterogeneity, task definition, reporting quality, and clinical integration [16,17,18]. Two methodological themes from this literature directly shape the present work. First, reported discrimination must be interpreted in light of how the reference standard was defined: when labels are derived from radiologist interpretation of the same examinations the model ingests, high area-under-the-curve values quantify agreement with the reading radiologist rather than accuracy against an independent standard such as arthroscopy. Second, on small single-center cohorts the apparent performance differences between closely matched configurations can be of the same order as cross-validation sampling variability, so such differences require paired statistical comparison, and any configuration chosen as the best of many should be treated as hypothesis-generating, before they are regarded as established. Both themes are addressed explicitly in the present study. A prior study in the literature evaluated patient-level rotator cuff tear classification on shoulder MRI using an explainable Vision Transformer framework, emphasizing patient-level aggregation and the anatomical plausibility of visual explanations [19].

However, one important question remains underdeveloped in the rotator cuff tear literature: how should different MRI sequences and non-image clinical variables be used together? Shoulder MRI is inherently multi-sequence. A conventional examination consists of a set of acquisitions that differ in contrast weighting, fat suppression, plane, anatomical emphasis, and susceptibility to sequence-specific artifacts, rather than a single homogeneous image series. T1-weighted coronal imaging may provide useful anatomical and marrow context; T2 fat-suppressed sagittal imaging is sensitive to fluid and muscle-tendon relationships; PD fat-suppressed coronal imaging is often informative for supraspinatus tendon integrity; and PD fat-suppressed axial or transverse imaging contributes transverse-plane information on the cuff, biceps, and related structures. From a machine learning perspective, these inputs may provide complementary information, but they can also introduce redundancy, domain heterogeneity, or weakly informative features that are amplified in small datasets.

The broader medical imaging literature provides a strong methodological basis for treating sequence selection as an empirical question rather than a fixed assumption. Multi-sequence and multiparametric MRI studies have shown that MRI sequences can contain both complementary and redundant information, and that complete protocols are not automatically optimal for every downstream learning objective [20,21,22,23,24,25,26,27,28,29,30,31]. In multi-sequence MRI representation learning, redundant information may interfere with efficient feature mining, while selected sequence subsets can sometimes approach full-protocol performance [20]. Missing-modality and incomplete-modality brain MRI studies similarly show that performance depends on which sequence is missing, which anatomical or pathological target is being predicted, and how fusion is performed [24,25,26,27,28,29]. The pediatric brain tumor segmentation work by Piffer et al. is relevant to the present study only as a conceptual example of this sequence-selection logic; because it concerns brain tumor segmentation rather than shoulder MRI or rotator cuff tear classification, it should be viewed strictly as a conceptual analogue rather than direct musculoskeletal evidence [21].

Clinical metadata adds a second layer of complexity. Age, sex, body habitus, laterality, and acquisition year can be clinically meaningful or technically informative, but such variables can also encode site-specific practice patterns, missingness, referral bias, or non-causal correlations. Reviews of image and electronic health record (EHR) fusion alongside multimodal biomedical AI suggest that structured clinical variables may improve imaging-based models when they add information not already present in the images. However, these reviews also warn that fusion design, missingness, confounding, and validation strategies ultimately determine whether a multimodal model is genuinely viable across diverse clinical scenarios [32,33,34,35,36,37,38,39,40]. In other words, metadata should not be treated as a universally beneficial attachment to an image model; its value must be rigorously tested in relation to the specific image feature set and task.

To address these challenges, we developed a multi-sequence, multimodal deep learning framework utilizing both convolutional (ResNet50) and transformer-based (Vision Transformer) architectures explicitly designed for patient-level rotator cuff tear diagnosis. Rather than presenting this framework as a static, unyielding configuration, we evaluate its behavioral mechanics through a systematic, hierarchical experimental design. Within this paradigm, “hierarchical” refers directly to the sequential decision flow of our investigations. Study 0 establishes single-sequence baselines and contrasts the inductive biases of ResNet50 against Vision Transformers under a unified, patient-level evaluation protocol. Study 1 benchmarks alternative multimodal fusion topologies using the complete four-sequence clinical protocol plus metadata, thereby isolating a highly stable fusion family suitable for combinatorial scaling. Study 2 then systematically maps all pairwise and three-sequence MRI combinations with and without metadata. This structural pipeline mirrors a practical clinical engineering question: before asserting that a multimodal system is ready for clinical deployment, one must determine which imaging sequence is intrinsically informative alone, which fusion strategy generalizes reliably, and whether adding more cross-domain data actually yields an incremental diagnostic benefit.

The core contributions of this study are therefore fourfold:

- First, we present a single-center, multi-sequence shoulder MRI cohort (n=199) accompanied by structured clinical metadata, explicitly curated for binary rotator cuff tear classification under a strict, leakage-free protocol.
- Second, we provide a comprehensive, slice-masked baseline benchmark comparing convolutional and transformer-based backbones across four distinct MRI planes and contrast weightings.
- Third, we systematically compare alternative multimodal fusion strategies to isolate the most robust decision-level archetype for multi-sequence aggregation.
- Fourth, we execute a granular sequence-combination analysis that isolates and quantifies the exact marginal performance deltas induced by clinical metadata across precisely matched feature spaces.

As a conclusion, our empirical evaluations demonstrate that the highest diagnostic performance within this cohort is achieved by a streamlined three-sequence subset (T2 + PDc + PDt) enhanced by clinical metadata, rather than by indiscriminately aggregating the entire clinical imaging protocol. This critical takeaway proves that adding non-fat-suppressed anatomical series introduces feature redundancy that degrades classification boundaries, showcasing that targeted sequence selection is essential for optimizing multimodal medical architectures.

## 2. Materials and Methods

This section delineates the methodological framework established to systematically evaluate multi-sequence shoulder MRI and clinical metadata fusion for rotator cuff tear classification. It describes the retrospective cohort definition, ethical considerations, and parameters governing multiplanar MRI acquisitions. Furthermore, it details the problem formulation, rigorous image preprocessing, slice standardization techniques, data augmentation strategies, and patient-level cross-validation protocols. Finally, it presents the specific deep learning architectures (ResNet50 and Vision Transformers), structured clinical metadata encoding pipelines, multimodal fusion strategies, optimization schedules, and visual explainability methodologies in accordance with transparent medical AI reporting standards.

**Figure 1:**
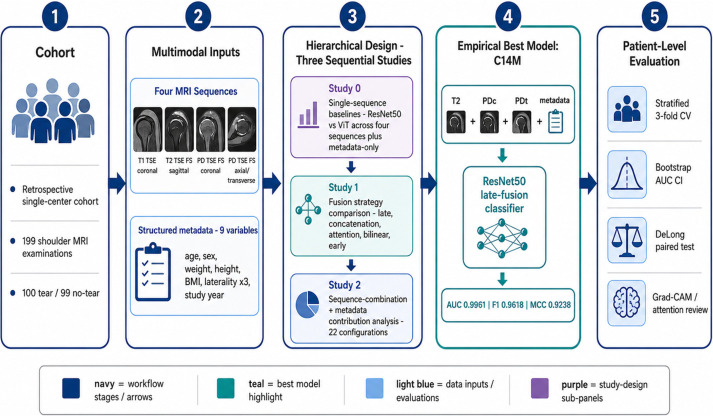
Schematic representation of the hierarchical multimodal workflow for patient-level rotator cuff tear classification. The experimental pipeline progresses systematically through five core operational stages: (1) retrospective assembly of the single-center cohort (n=199), comprising balanced tear and no-tear cases; (2) multiplanar ingestion of four distinct MRI sequence weightings alongside a 9-variable normalized clinical metadata vector; (3) execution of the hierarchical design divided into three sequential investigations (Studies 0, 1, and 2); (4) empirical isolation and deployment of the optimized ResNet50 late-fusion classifier (C14M); and (5) rigorous multi-metric patient-level evaluation leveraging stratified three-fold cross-validation, bootstrapping, paired DeLong testing, and Grad-CAM/attention-driven visual quality control maps. CV: cross-validation, CI: confidence interval, AUC: Area Under the ROC Curve, TSE: turbo spin-echo, PD: proton density, FS: fat-suppressed.

The global experimental architecture and the five-stage procedural flow of this study are illustrated in Figure 1. To preserve a clear structural connection between the subheadings and the operational flow depicted in the schematic, detailed descriptions of these stages are organized and given as follows:

- Stage 1 (Cohort Assembly): Detailed patient baseline and ethical parameters are provided in Section 2.1.
- Stage 2 (Multimodal Inputs): Technical MRI acquisition profiles and structured metadata encoding pipelines are described in Section 2.2 and Section 2.3.
- Stage 3 (Hierarchical Design): Problem formulation, standardization, image preprocessing, and core model architectures are mapped across Section 2.4, Section 2.5, Section 2.7, Section 2.8 and Section 2.11.
- Stage 4 (Empirical Best Model Topology): Multimodal fusion strategies, regularization schedules, and optimization details are evaluated in Section 2.9 and Section 2.10.
- Stage 5 (Patient-Level Evaluation): Cross-validation protocols, statistical output metrics, explainability mapping, and reporting standards are presented in Section 2.6, Section 2.12, Section 2.13 and Section 2.14.

### 2.1. Study Design, Ethical Context, and Cohort Definition

This retrospective, single-center study included 199 patients who underwent shoulder MRI and had binary labels for rotator cuff tear classification. The positive class was defined as the presence of a rotator cuff tear, and the negative class was defined as the absence of a tear. The cohort comprised 100 tear cases and 99 no-tear cases. The mean age of the cohort was 50.2±18.1 years, and the mean body mass index (BMI) was $26.5\pm 3.2{\;\mathrm{kg}/m}^{2}$. The cohort included 118 female and 81 male patients. Laterality was right-sided in 74 patients, left-sided in 56 patients, and unspecified in 69 patients. MRI examinations were performed in either 2024 (n=157) or 2025 (n=42). Detailed demographic and clinical characteristics are provided in Table 1.

The study was designed as a patient-level diagnostic modeling study. To prevent data leakage arising from slice-level correlation, all slices belonging to a single patient were strictly confined to the same cross-validation fold. This partitioning strategy is critical because shoulder MRI examinations contain multiple adjacent slices from the same anatomical volume; if slices from the same patient appear across both training and test partitions, model performance metrics may be artificially inflated.

Regarding the reference standard, the binary tear/no-tear labels used in this study were obtained from the patients’ institutional clinical records and were confirmed by the orthopedic surgery and radiology specialist co-authors. They therefore constitute an expert-confirmed institutional clinical reference standard derived from routine specialist interpretation of the same shoulder MRI examinations that serve as the model input; the labels were not independently validated against arthroscopic or surgical findings. Consequently, the performance metrics reported in this work quantify the framework’s concordance with expert clinical MRI reads rather than its accuracy against an independent, image-external gold standard. This distinction should be borne in mind when interpreting the high discrimination values, and it motivates the use of “classification” rather than “diagnosis” terminology throughout the present work.

The study was conducted in accordance with the Declaration of Helsinki. The research protocol, entitled “Multimodal Diagnosis and Virtual Assistant Use in Magnetic Resonance Imaging for Rotator Cuff Tear Diagnosis,” was reviewed and approved by the Non-Interventional Clinical Research Ethics Committee of Bilecik Şeyh Edebali University (Decision No. 9, 10th Meeting, dated 30 October 2025).

### 2.2. MRI Acquisition and Sequence-Level Inputs

For each patient, four distinct MRI sequences were evaluated: T1-weighted turbo spin-echo (TSE) coronal, T2-weighted TSE fat-suppressed sagittal, proton density (PD)-weighted TSE fat-suppressed coronal, and PD-weighted TSE fat-suppressed axial/transverse imaging. Acquisition metadata indicated a 256×256 image matrix across all four sequence groups. The mean repetition time (TR) and echo time (TE) were approximately 669.2/20.1 ms for T1 TSE coronal, 4258.2/71.0 ms for T2 TSE fat-suppressed sagittal, 3115.7/34.1 ms for PD TSE fat-suppressed coronal, and 3137.3/26.0 ms for PD TSE fat-suppressed axial/transverse imaging. The mean slice thickness was approximately 4.3 mm across all sequences. These acquisition parameters are summarized in Table 2.

For concise reporting, these sequences are abbreviated throughout the manuscript as T1, T2, PDc, and PDt, respectively. The distinction between sequence identity (contrast weighting) and anatomical plane was rigorously preserved throughout the analysis, as deep learning models can exploit both contrast-specific and plane-specific features. Consequently, the sequence-combination analysis treats each sequence-plane acquisition as a distinct, independent input channel at the examination level, rather than assuming that all MRI series are interchangeable.

### 2.3. Structured Clinical Metadata

Structured clinical metadata included age, sex, weight, height, BMI, laterality, and acquisition study year. The metadata pipeline ingested nine numeric inputs: age, sex, weight (in kilograms), height (in meters), BMI, right laterality, left laterality, unspecified laterality, and study year. Continuous variables were scaled using min-max normalization, where parameters were fitted exclusively on the training set within each fold and subsequently applied to the corresponding validation and test sets. Laterality was one-hot encoded into three dimensions (right, left, or unspecified). This fold-specific preprocessing strategy was strictly enforced to prevent validation or test information from leaking into the training pipeline.

Metadata was treated as a distinct, separate modality rather than a surrogate for imaging. A metadata-only model baseline was established to quantify the baseline diagnostic signal that could be extracted from structured variables alone. In multimodal configurations, the clinical metadata vector was encoded by a small multilayer perceptron (MLP) and either concatenated with image-derived features or converted into an independent decision component, depending on the fusion architecture. This design permitted an empirical evaluation of whether metadata improves specific sequence combinations, rather than merely reporting a single global image-plus-metadata result.

### 2.4. Problem Formulation

The diagnostic task was formulated as a patient-level binary classification problem leveraging a set of sequence-specific MRI stacks and a structured metadata vector. For patient i, the multi-sequence MRI input is represented as:

(1) $$X_{i}=\left\{I_{i}^{s}|s\in S_{i}\right\},$$

where s denotes an individual MRI sequence from the sequence set $S_{i}$, and $I_{i}^{s}=\left\{x_{i,j}^{s}\right\}$ for $j=1,\ldots ,N_{i}^{s}$ denotes the chronologically ordered stack of slices for that specific sequence s. Each individual slice $x_{i,j}^{s}$ is a two-dimensional grayscale MRI image resized and formatted as a three-channel tensor to ensure compatibility with ImageNet-pretrained backbones. The patient-level ground-truth label is defined as $y_{i}\in \left\{0,1\right\}$, where 1 denotes a rotator cuff tear and 0 denotes no tear. The structured clinical features are represented as a metadata vector $m_{i}\in R^{9}$.

The optimization objective is to learn a mapping $F\left(X_{i},m_{i}\right)\to p_{i}$, where $p_{i}$ is the predicted probability of a rotator cuff tear.

- In Study 0, $X_{i}$ contains only a single sequence, and the model estimates $p_{i}$ from a single image stack or from the metadata vector alone.
- In Study 1, $X_{i}$ encompasses all four MRI sequences alongside metadata to determine the optimal architectural fusion strategy.
- In Study 2, $X_{i}$ comprises a systematically selected subset of two, three, or four MRI sequences evaluated across matched metadata-free and metadata-enhanced variants. This formulation treats the sequence selection configuration itself as an explicit experimental variable.

### 2.5. Image Loading, Normalization, and Slice Standardization

MRI slices were parsed using a slice-level metadata index that mapped patient identifiers, binary labels, sequence definitions, instance numbers, and image file paths. For each patient and sequence, slices were sorted by instance number to preserve true anatomical ordering. Images were normalized and resized to 224×224 pixels using bilinear interpolation. To satisfy fixed-length batch requirements, each sequence stack was standardized to exactly 20 slices via center-padding or center-trimming. Center-trimming was executed if the original stack exceeded 20 slices, while symmetric zero-padding was applied if the stack contained fewer than 20 slices. A binary slice validity mask was retained to differentiate real anatomical images from padded positions.

This slice mask is central to the pipeline’s engineering implementation. Let $h_{i,j}^{s}$ be the feature vector extracted from slice j of sequence s, and let $a_{i,j}^{s}$ be the corresponding binary validity indicator ($a_{i,j}^{s}=1$ for real slices, 0 for padded frames). The aggregated sequence-level representation $z_{i}^{s}$ was computed via masked averaging:

(2) $$z_{i}^{s}=\frac{\sum_{j}a_{i,j}^{s}h_{i,j}^{s}}{\max\left(\sum_{j}a_{i,j}^{s},1\right)},$$

This masking formulation prevents zero-padded slices from corrupting the sequence-level representation and allows a uniform architecture to process variable slice counts within a batch.

During training, data augmentation was applied exclusively to valid anatomical slices. The augmentation pipeline included horizontal flipping (probability = 0.5), random rotation (±10°), spatial scaling (0.9 to 1.1), shear transforms (up to 5°), intensity shifting (up to 0.1), and the injection of Gaussian noise (σ=0.01). Augmentations were restricted entirely to the training sets and were never applied to validation or test folds.

### 2.6. Patient-Level Cross-Validation and Data Loading

All experiments were evaluated using a stratified three-fold outer cross-validation scheme initialized with random seed 42. Within each outer split, a stratified inner validation partition comprising 15% of the training pool was utilized for early stopping and hyperparameter optimization. The held-out test folds contained 67, 66, and 66 patients, respectively, ensuring that all 199 patients were evaluated exactly once across the three outer folds. The target class was the presence of a rotator cuff tear. Identical fold boundaries were maintained across all model families and sequence combinations to ensure paired, statistically rigorous performance comparisons.

Class imbalances were addressed at both the data-loading and loss-function levels. Image models integrated a weighted random sampler wherein individual training samples were weighted inversely to their respective class frequencies within that specific training fold. Furthermore, the positive-class weight term for the binary cross-entropy loss function was dynamically calculated per training fold as the exact ratio of negative to positive cases. These dual mitigation strategies were implemented because small, fold-level class variations can destabilize decision thresholds, particularly when optimizing high-dimensional imaging models on relatively small patient cohorts.

### 2.7. Backbone Encoders and Patient-Level Representations

The primary convolutional neural network backbone evaluated was an ImageNet-pretrained ResNet50 architecture [41]. For each slice, the final fully connected classification layer was removed, enabling the convolutional feature extractor to yield a 2048-dimensional feature vector. For a given MRI sequence, slice-level features were aggregated using the previously defined masked mean pooling to derive a singular sequence-level vector. Single-sequence ResNet variants utilized this vector directly (or concatenated it with metadata representations when applicable). Multi-sequence ResNet architectures dedicated an independent sequence encoder to each respective MRI series, subsequently merging the sequence-level vectors according to the specified fusion paradigm.

The Vision Transformer comparator leveraged a pretrained vit_base_patch16_224 backbone [42]. Each 224×224 pixel slice was tokenized into 16×16 patches, projected into a 768-dimensional embedding space, and processed through the transformer blocks. To construct a patient-level representation, individual slice embeddings were passed through a temporal sequence-encoding module and aggregated using a class-token ([CLS]) representation. Sequence-specific learnable embeddings were integrated to preserve MRI sequence identity when multiple sequences were processed simultaneously. The implementation adheres to established deep learning guidelines in medical imaging AI, including transfer learning, strict patient-level validation tracking, and reproducible reporting of preprocessing configurations [43,44]. The Vision Transformer models serve exclusively as a comparator framework in Study 0 and Study 1 rather than being presented as a novel architectural contribution.

### 2.8. Metadata Encoder and Classification Heads

The metadata encoder consisted of a two-layer MLP. The raw nine-dimensional metadata vector was projected to a 64-dimensional hidden layer, followed by layer normalization and a Gaussian Error Linear Unit (GELU) activation function, and then projected to a final 128-dimensional metadata representation with an additional layer normalization and GELU activation. The standalone metadata baseline model utilized a separate MLP architecture configured with hidden dimensions of 64 and 32, layer normalization, GELU activations, a dropout rate of 0.3, and a final scalar logit output.

Image-based classification heads were constructed using fully connected layers combined with layer normalization, GELU activations, and dropout regularizers. The default classification head projected fused representations through hidden dimensions of 256 and 64 prior to computing the final binary logit. For models executing explicit feature concatenation across multiple parallel sequences, an expanded classification head with hidden dimensions of 512 and 128 was utilized to accommodate the substantially increased input feature dimensionality. This architectural adaptation is necessary because feature concatenation preserves high-dimensional joint spaces, whereas late decision fusion merges low-dimensional scalar logits, thereby requiring fewer trainable parameters at the final fusion layer.

### 2.9. Multimodal Fusion Strategies

Study 1 systematically compared several fusion methodologies leveraging all four MRI sequences alongside clinical metadata.

- Late decision-level fusion first generated independent logits for each individual MRI sequence and, where applicable, a separate logit from the clinical metadata vector. The unified final logit $l_{i}$ was computed as a learned, softmax-weighted combination:
  (3) $$l_{i}=\sum_{k}\alpha_{k}l_{i,k},$$
  where the attention vector α is derived via α=softmax(w), and k indexes the respective sequence and metadata pathways. This configuration isolates modality-specific decision pathways and delays interaction until the logit stage.
- Feature concatenation fused sequence-level image representations prior to the classification layers. For a selected sequence subset S, the fused joint vector $z_{i}$ was formed as:
  (4) $$z_{i}=\left[z_{i}^{s_{1}};z_{i}^{s_{2}};\ldots ;z_{i}^{s_{n}};u_{i}\right],$$
  where $u_{i}$ denotes the encoded metadata vector. This strategy allows multi-sequence feature interactions within the classification head but scales up dimensionality, increasing vulnerability to overfitting on constrained datasets.
- Attention-weighted fusion dynamically learned sequence importances from projected feature states using a tanh transformation coupled with a scalar attention scoring mechanism followed by softmax normalization across sequences.
- Compact bilinear pooling iteratively fused sequence vectors through randomized count-sketch projections and elementwise multiplication in the Fourier domain, finalized by signed square-root and $L_{2}$ normalizations.
- Early fusion was restricted to structurally compatible paired sequences and concatenated raw image channels prior to ResNet encoding, utilizing a modified six-channel first convolutional layer adapted from the pretrained three-channel weights.

The purpose of these evaluations was to select a highly stable and performant fusion archetype for the subsequent sequence-combination experiments. Late fusion was selected as the core paradigm for Study 2 because it yielded the highest Area Under the Receiver Operating Characteristic curve (ROC-AUC) in Study 1 and exhibited excellent cross-metric stability. The competitive F1-score performance of the feature concatenation architecture was retained during subsequent interpretation to ensure multi-metric validation.

### 2.10. Optimization, Regularization, and Reproducibility

All deep learning models were trained for a maximum of 30 epochs with an early stopping patience of 7 epochs. To reduce the risk of selecting an epoch with an inflated AUC but poor threshold-dependent behavior, early stopping was governed by a composite validation score:

(5) Score=0.6×AUC+0.4×F1-score,

Label smoothing (ϵ=0.1) was applied during training. The objective function was minimized using binary cross-entropy with logits, integrating the fold-specific positive-class weights.

Optimization was driven by the AdamW optimizer paired with a weight decay of $1\times 10^{-4}$ and a cosine annealing warm-restart learning rate scheduler. ResNet models utilized a backbone learning rate of $1\times 10^{-4}$ and a classification head learning rate of $1\times 10^{-3}$. Vision Transformer variants utilized a backbone learning rate of $1\times 10^{-5}$ and a head learning rate of $1\times 10^{-4}$. ResNet models were trained with a batch size of 8 and a gradient accumulation step of 2, whereas Vision Transformer models used a batch size of 4 and a gradient accumulation step of 4. Model backbones were frozen for the first 2 epochs for ResNet and the first 3 epochs for Vision Transformers. Gradient clipping with a maximum norm of 1.0 was enforced. All cross-validation experiments were explicitly seeded at execution initialization, and deterministic cuDNN execution flags were set to ensure pipeline reproducibility.

### 2.11. Hierarchical Experimental Design

The research pipeline was partitioned into three sequential, hierarchical experimental blocks, summarized in Table 3.

- Study 0 evaluated each individual MRI sequence independently using both ResNet50 and Vision Transformer backbones alongside the standalone metadata MLP baseline. This block established which singular imaging sequence retained the highest intrinsic diagnostic signal and verified whether the transformer architecture provided an advantage over the convolutional baseline on this cohort size.
- Study 1 comprehensively evaluated multimodal fusion strategies using all four MRI sequences combined with clinical metadata. Competing methods included ResNet late decision-level fusion, feature concatenation, attention-weighted fusion, compact bilinear pooling, early channel fusion, and parallel Vision Transformer fusion variants. This block determined the optimal fusion architecture to carry forward into the sequence subset analysis.
- Study 2 executed a systematic evaluation of partial MRI sequence combinations with and without clinical metadata. Because single-sequence models were thoroughly charted in Study 0, Study 2 focused specifically on all possible pairwise, three-sequence, and full four-sequence permutations. The incremental value of clinical data was strictly isolated and quantified as the performance delta (ΔAUC, Δprecision, Δsensitivity, and ΔF1-score) between precisely matched metadata-enhanced and metadata-free configurations.

### 2.12. Performance Metrics, Confidence Intervals, and Statistical Outputs

Model classification performance was evaluated across the three independent patient-level test folds. Computed evaluation metrics included ROC-AUC, average precision (AP), accuracy, sensitivity, specificity, precision, F1-score, the Matthews correlation coefficient (MCC), negative predictive value (NPV), and cross-entropy loss. Threshold-dependent metrics were computed using a fixed decision threshold of 0.5. Specificity was calculated as TN/(TN+FP), sensitivity as TP/(TP+FN), precision as TP/(TP+FP), and NPV as TN/(TN+FN). The MCC was tracked to provide a balanced assessment of binary classification quality that remains robust against fold-level variations in class proportions.

Confidence intervals for the ROC-AUC metrics were generated via a percentile bootstrap (fixed seed) over the pooled out-of-fold predictions, using 2000 resamples. Paired ROC-AUC statistical significances were computed using DeLong’s test [45]. Model selection criteria avoided reliance on any singular performance metric; instead, the final selection integrated ROC-AUC, F1-score, sensitivity/specificity balance, precision, MCC stability, and the variance of confidence intervals across the outer validation folds. For the metadata-contribution analysis (Section 3.5), 95% confidence intervals on the paired performance deltas were additionally estimated with a fold-matched paired bootstrap (2000 resamples, fixed seed), in which patient indices were resampled within each cross-validation fold so that the fold structure was preserved and the two compared configurations were evaluated on identical resampled patients. Because the top-ranked Study 2 configuration was identified post hoc across all 22 sequence/metadata configurations evaluated on the same outer folds, the between-configuration comparisons reported here are descriptive and hypothesis-generating rather than confirmatory; nested cross-validation, in which the configuration choice is made entirely within the training folds, is identified as future work.

### 2.13. Explainability and Visual Quality Control

Experimental outputs generated by the evaluation pipeline included ROC curves, confusion matrices, fold-level prediction spreads, and explainability galleries. For ResNet-based models, Gradient-weighted Class Activation Mapping (Grad-CAM) visualizations were computed from the final feature maps of the terminal convolutional layer [46]. For Vision Transformer comparators, attention-based saliency maps were extracted from the self-attention weights. To prevent heatmaps from misallocating importance to uninformative zero-padded background matrices or air artifacts outside the anatomical boundaries, a binary foreground mask was derived directly from the source image and applied to the computed saliency maps. These explainability outputs were interpreted strictly as qualitative architectural checks rather than clinical proof of human-like radiological reasoning.

The primary visual evidence compiled for manuscript reporting consists of the hierarchical experimental workflow diagram, the Study 0 sequence baseline matrix, the Study 1 fusion-strategy comparison chart, the Study 2 sequence-combination performance heatmap, the metadata contribution delta plot, and the comprehensive diagnostic performance panel. While explainability maps offer qualitative support for anatomical plausibility, the core scientific findings of this study rely strictly on patient-level quantitative test fold data.

### 2.14. Reporting Standards

The structure and reporting of this manuscript align strictly with established medical imaging AI guidelines, including transparent cohort assembly criteria, explicit patient-level split tracking, precise task definitions, fold-specific metric reporting, and a clear disclosure of external validation constraints. The CLAIM (Clinical AI Modeling), TRIPOD+AI, and STARD-AI reporting frameworks were utilized as foundational checklists for diagnostic accuracy and medical AI study execution [47,48,49]. Accordingly, this study should be interpreted as a rigorous model-development and internal-validation effort, rather than a clinical trial of a deployed clinical decision-support system.

## 3. Results

In this section, the empirical findings derived from the hierarchical experimental framework are systematically presented across three distinct analytical phases. First, the demographic and acquisition baseline characteristics of the 199-patient cohort are outlined. Next, the unimodal diagnostic capabilities of the deep convolutional and transformer-based networks are established alongside a standalone clinical metadata baseline (Study 0). This is followed by a comprehensive architectural comparison of alternative multimodal fusion topologies using the full four-sequence clinical protocol (Study 1). Finally, a systematic sequence-combination analysis isolates the optimal partial imaging configurations and quantifies the performance deltas induced by clinical metadata integration (Study 2), alongside cross-validation stability and qualitative explainability assessments.

### 3.1. Cohort and MRI Inputs

The final cohort included 199 patients, consisting of 100 tear cases and 99 no-tear cases. All patients had the four predefined shoulder MRI sequences available: T1, T2, PDc, and PDt. The three held-out test folds contained 67, 66, and 66 patients, respectively. The balanced target class distribution allowed sensitivity and specificity to be interpreted directly without the skew of a majority-class imbalance. However, fold-level variance remained experimentally relevant because each validation partition contained approximately one-third of the total cohort.

The demographic and clinical characteristics are summarized in Table 1, and the acquisition parameters are detailed in Table 2. The sequence abbreviations used throughout this section correspond directly to the acquisition groups defined in the methods. The sequence-combination alphanumeric labels in Study 2 follow a systematic configuration matrix, where the presence or absence of each individual MRI sequence is indexed relative to the baseline order (T1, T2, PDc, and PDt). The suffix “M” explicitly denotes the inclusion of structured clinical metadata.

### 3.2. Study 0: Single-Sequence Baselines and ResNet-ViT Comparison

Study 0 demonstrated that individual MRI sequences contained a highly robust diagnostic signal for binary rotator cuff tear classification. Among all single-sequence models evaluated, the convolutional ResNet50 baseline trained on the PDc sequence yielded the highest overall diagnostic performance, achieving an AUC=0.9834, accuracy=0.9498, sensitivity=0.9700, specificity=0.9293, F1-score=0.9515, a Matthews correlation coefficient (MCC) of 0.9028, and a bootstrapped 95% confidence interval (CI) for the AUC of 0.9508–0.9930. The ResNet T2 model also performed strongly (AUC=0.9780; F1-score=0.9110), followed closely by the ResNet PDt model (AUC=0.9742; F1-score=0.9064). These findings are clinically plausible, as proton density fat-suppressed coronal imaging and fluid-sensitive sagittal views are the radiological standards for evaluating tendon integrity, fluid-tendon interfaces, and general muscle-tendon relationships.

The single-sequence Vision Transformer (ViT) architectures were highly competitive but did not outperform the top-performing convolutional baselines on this dataset. The highest performing ViT configuration was achieved using the PDt sequence (AUC=0.9663; F1-score=0.9106), followed by T2 (AUC=0.9594; F1-score=0.9388) and PDc (AUC=0.9564; F1-score=0.9002). This performance gap does not imply that transformer models are inherently unsuited for shoulder pathodynamics; rather, it indicates that given our 199-patient cohort size, the ImageNet-pretrained ResNet50 encoder combined with our masked slice-level aggregation and fold-level optimization pipeline yielded a superior single-sequence diagnostic profile.

The standalone clinical metadata model performed substantially worse than all image-based models, yielding an AUC=0.6104 and an F1-score=0.6575. This baseline is important because it prevents the overinterpretation of clinical risk factors; while non-image electronic health record variables hold an undercurrent of diagnostic signal, they are wholly insufficient for accurate classification without corresponding multiplanar imaging features. Thus, Study 0 justified two fundamental downstream choices: deep image feature mining is mandatory for this task, and the ResNet backbone offers the most stable baseline for the subsequent combinatorial analysis. The complete single-sequence baseline performance metrics are documented in Table 4.

### 3.3. Study 1: Multimodal Fusion Strategy Comparison

Study 1 compared multimodal fusion architectures leveraging the complete four-sequence MRI protocol alongside clinical metadata. The ResNet late-fusion architecture achieved the highest ranking capacity among all evaluated options, demonstrating an AUC=0.9909, accuracy=0.9346, sensitivity=0.9495, specificity=0.9192, precision=0.9236, F1-score=0.9355, MCC=0.8715, and an AUC 95% CI of 0.9699–0.9974. Conversely, the ResNet feature-concatenation model achieved a lower overall area under the curve but produced a higher localized F1-measure (AUC=0.9753; F1-score=0.9502; MCC=0.9030). The late-fusion Vision Transformer model yielded an AUC=0.9722 and an F1-score=0.9237.

These discrepancies emphasize why multimodal optimization should never be collapsed into a single metric. Late fusion optimized global ranking accuracy and achieved a balanced sensitivity-specificity profile across thresholds, whereas feature concatenation maximized the explicit precision-recall trade-off at the default 0.5 classification threshold. In a clinical diagnostic pipeline, both characteristics are valuable: superior ranking performance supports custom operational threshold tuning, while the F1-score reflects the real-world performance of an uncalibrated, deployed model.

ResNet late fusion was chosen as the primary engine for Study 2 due to its superior AUC profile in Study 1 and its modular, decision-level configuration. By isolating each sequence’s logit contribution prior to weighted aggregation, the late-fusion pipeline dampens the risk of high-dimensional concatenated spaces causing catastrophic overfitting on constrained training partitions. The comparison of these distinct multimodal architectures is detailed in Table 5.

### 3.4. Study 2: MRI Sequence-Combination Analysis

Study 2 investigated whether optimal diagnostics require the full diagnostic sequence complement, or if selective subset configurations could yield superior results. The top-performing overall model across the entire experimental block was C14M, which combined the T2, PDc, and PDt sequences with structured clinical metadata. C14M achieved an AUC=0.9961, accuracy=0.9600, sensitivity=0.9798, specificity=0.9394, precision=0.9475, F1-score=0.9618, MCC=0.9238, a negative predictive value (NPV) of 0.9810, and a tight bootstrapped 95% CI for the AUC of 0.9823–0.9987. The comprehensive evaluation of all combinatorial variations with and without clinical metadata is structured in Table 6.

The fold-level performance metrics for the optimal C14M configuration were highly consistent across the cross-validation splits, as detailed below:

- Fold 0: AUC=0.9929, accuracy=0.9254, sensitivity=1.0000, specificity=0.8485, precision=0.8718, F1-score=0.9315.
- Fold 1: AUC=0.9991, accuracy=0.9848, sensitivity=1.0000, specificity=0.9697, precision=0.9706, F1-score=0.9851.
- Fold 2: AUC=0.9963, accuracy=0.9697, sensitivity=0.9394, specificity=1.0000, precision=1.0000, F1-score=0.9688.

This breakdown demonstrates excellent ranking stability across all validation partitions, with minor localized fluctuations confined to threshold-dependent sensitivity and specificity trade-offs. These threshold-dependent swings are expected at this sample size: each held-out fold contains only about 33 tear and 33 non-tear patients, so a single additional false negative or false positive shifts that fold’s sensitivity or specificity by roughly one in 33 (≈3 percentage points) and fold-level accuracy by about one in 66 (≈1.5 percentage points). The fold-0 specificity of 0.8485, for example, corresponds to 28 of 33 true negatives, and one additional correct classification would raise it to 0.879. Per-fold sensitivity and specificity should therefore be read as coarse-grained estimates, with the bootstrap confidence intervals providing the more stable summary. The detailed cross-validation slice tracking for the optimal C14M framework is reported in Table 7.

Crucially, the full four-sequence metadata-enhanced configuration was outperformed by the streamlined three-sequence C14M model, with the complete protocol model achieving a lower AUC=0.9909, accuracy=0.9346, sensitivity=0.9495, specificity=0.9192, precision=0.9236, F1-score=0.9355, and MCC=0.8715. In this cohort, excluding the T1 sequence while preserving the fat-suppressed, fluid-sensitive, and proton density sequences maximized classification accuracy. This does not imply that T1-weighted sequences are clinically redundant; rather, it highlights that for this specific binary computer-aided diagnosis task, adding non-fat-suppressed anatomical data introduced feature noise that detracted from the core signal captured by the remaining modalities.

### 3.5. Metadata Contribution

The diagnostic contribution of clinical metadata was highly dependent on the baseline image sequence combination. The most pronounced positive effect occurred when metadata was integrated into the three-sequence C14 combination (T2 + PDc + PDt). Upgrading from C14 to C14M increased the performance profile substantially: ΔAUC=+0.0262, Δprecision=+0.0447, Δsensitivity=+0.0909, and ΔF1-score=+0.0690. Modest positive improvements were also visible in other subsets, such as the transition from C09 to C09M (ΔAUC=+0.0093; ΔF1-score=+0.0264) and C10 to C10M (ΔAUC=+0.0067; ΔF1-score=+0.0127).

However, the addition of metadata was not a universal benefit. For certain configurations, metadata injection damaged model execution; for example, upgrading C13 to C13M resulted in a performance drop (ΔAUC=−0.0081; ΔF1-score=−0.0127), and the transition from C05 to C05M similarly degraded performance (ΔAUC=−0.0187; ΔF1-score=−0.0265).

To distinguish genuine effects from fold-level fluctuation, paired (fold-matched) bootstrap 95% confidence intervals were computed for every delta (Table 8 footnote). Only the headline upgrade C14 → C14M produced a ΔAUC whose interval clearly excluded zero (+0.0158 to +0.0640); the smaller C09 → C09M and C10 → C10M gains were also reproducibly positive, while the C05 → C05M and C13 → C13M changes were reproducibly negative. The remaining deltas had intervals spanning zero and are best read as directionally suggestive but within cross-validation variability. The narrative below should therefore be interpreted as hypothesis-generating rather than as evidence of uniformly significant metadata effects. These trends strongly challenge the simplistic assumption that appending structured patient features always yields a net positive effect. Clinical data provided its greatest utility when paired with a highly informative image feature set (T2 + PDc + PDt), suggesting that demographic attributes stabilize or calibrate predictions only when the underlying visual tokens are already well-aligned with the pathology. When the imaging features are suboptimal or unaligned, appending clinical data introduces confounding parameters, reinforces site-specific biases, or skews classification thresholds unfavorably. This mirrors broader multimodal EHR-imaging trends where the utility of clinical variables is bounded by cross-modality complementarity and validation architecture [32,33,34,35,36,37,38,39,40]. The isolated performance differences detailing the specific positive and negative impacts of metadata integration are cataloged in Table 8.

### 3.6. Visual and Explainability Outputs

The generated experimental figures complement the quantitative tables by illustrating the hierarchical framework flow, sequence-combination performance heatmaps, metadata contribution delta charts, and the final diagnostic performance profiles of the optimal configuration. The sequence-combination matrix heatmap is central to our core thesis, visually demonstrating that maximum performance plateaued before the integration of the full four-sequence imaging protocol. The metadata delta plot provides critical non-imaging context, showing the structural divergence between performance gains and losses based on the underlying image combination.

**Figure 2:**
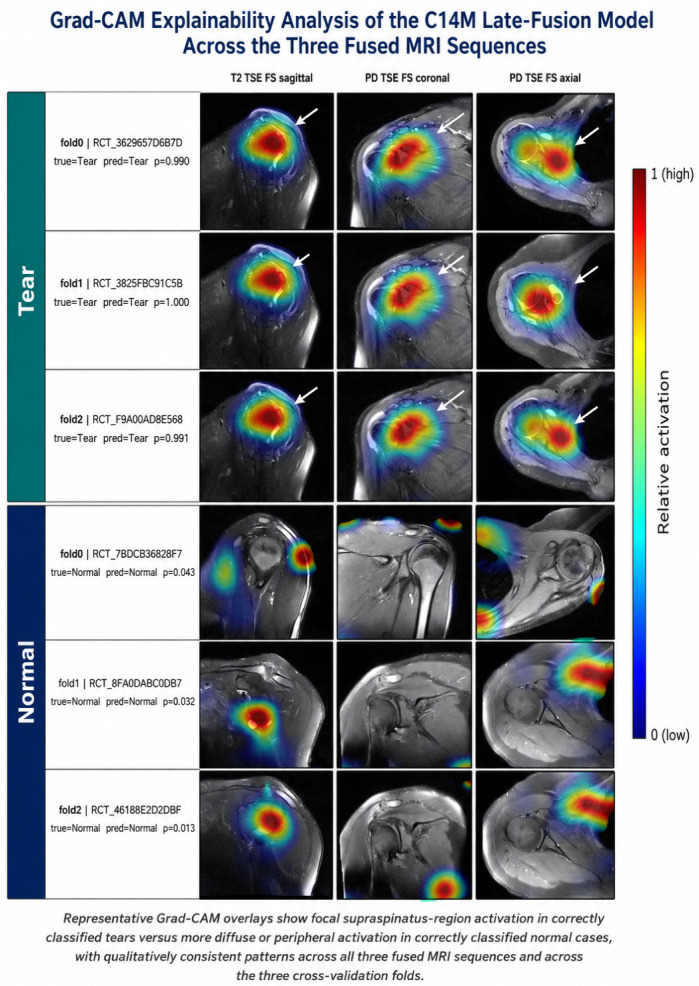
Out-of-fold Grad-CAM explainability matrix for the task-optimized late-fusion model (C14M) across the three fused MRI sequences (T2 sagittal, PD coronal, and PD axial). Rows are stratified by true patient pathology (Tear vs. Normal) across independent validation splits (folds 0, 1, and 2), with anonymized patient identifiers and true/predicted classes indicated. White arrows highlight the localized, highly focused supraspinatus-region activation in true tear cases, contrasting with the diffuse or peripheral background activation patterns observed in true normal/negative controls. The color bar represents relative feature activation maps normalized from 0 (low) to 1 (high). TSE: turbo spin-echo, FS: fat-suppressed, PD: proton density.

Saliency maps were treated strictly as conservative visual quality metrics. While Grad-CAM heatmaps and ViT attention maps allowed us to confirm that feature extraction localized to valid shoulder structures rather than zero-padded backgrounds or out-of-field air artifacts, these maps do not constitute proof of clinical reasoning, mechanistic understanding, or out-of-distribution generalizability. Representative out-of-fold Grad-CAM activation overlays across the cross-validation splits and fused imaging planes for the optimal late-fusion configuration (C14M) are systematically detailed in Figure 2. Consequently, these visualizations serve strictly as qualitative checks for anatomical plausibility, leaving the patient-level quantitative cross-validation data as the primary empirical validation of this study.

## 4. Discussion

This section contextualizes the empirical findings of our hierarchical framework within the broader landscape of musculoskeletal artificial intelligence and multiplanar medical imaging translation. We interpret the primary findings, highlighting the robust unimodal signal of fat-suppressed sequences and the nuanced paradox of modality redundancy where streamlined sequence subsets can optimize diagnostic accuracy over full clinical protocols. The role of structured clinical variables is examined as a context-dependent calibration signal rather than an independent predictor. Furthermore, the technical and engineering choices underpinning late decision fusion are critically evaluated alongside direct clinical implications for high-throughput computer-aided diagnosis (CADx) systems. Finally, the intrinsic limitations of this single-center, binary classification study are transparently disclosed, establishing a concrete roadmap for future multi-institutional validation and multi-task algorithmic development.

### 4.1. Principal Findings

This study evaluated patient-level binary rotator cuff tear classification using a hierarchical multimodal framework designed to systematically isolate the diagnostic value of single-sequence baselines, alternative fusion strategies, and multi-sequence MRI combinations coupled with structured clinical metadata. Three primary findings emerged from this empirical evaluation:

1. Robust Unimodal Signal: Individual MRI sequences—most notably proton density fat-suppressed coronal (PDc) and T2-weighted fat-suppressed sagittal (T2) series—contain a highly robust intrinsic diagnostic signal. The top-performing single-sequence model was the ResNet50 baseline trained on PDc images, achieving an AUC=0.9834 and an F1-score=0.9515.
2. Trade-offs in Multimodal Fusion: The optimal multimodal architecture depends heavily on fusion topology and metric prioritization. In our all-sequence baseline comparisons, late decision-level fusion maximized global ranking capacity (AUC=0.9909), whereas feature-level concatenation yielded a superior threshold-dependent precision-recall balance (F1-score=0.9502).
3. The Paradox of Modality Redundancy: Maximizing the volume of raw image data does not automatically improve diagnostic accuracy. The strongest overall diagnostic profile in this study was achieved by the C14M configuration—a streamlined three-sequence subset (T2 + PDc + PDt) enhanced by clinical metadata—which achieved the highest overall AUC and ranked above the full four-sequence metadata enhanced protocol; as detailed in Section 3.4, this difference did not reach statistical significance and is therefore reported as hypothesis-generating rather than as an established superiority.

Consequently, the central paradigm shift demonstrated by this study moves from an indiscriminate modality-maximal approach to a targeted sequence-aware optimization framework. Multimodal fusion delivers its greatest utility when constituent inputs provide distinct, complementary features regarding the underlying pathology. In our cohort, combining fluid-sensitive sagittal data, coronal tendon details, axial transverse context, and structured patient demographics provided a comprehensive diagnostic landscape. Adding further non-fat-suppressed anatomical series failed to add actionable incremental signals and instead introduced confounding parameters that degraded the model’s threshold-dependent classification metrics.

### 4.2. Relationship to Prior Shoulder MRI AI Studies

Contemporary deep learning literature has firmly established the feasibility of automated shoulder MRI interpretation, demonstrating excellent performance across tasks such as rotator cuff tear detection, supraspinatus tear grading, muscle segmentation, and the quantification of fatty infiltration [7,8,9,10,11,12,13,14,15,16,17,18,19,50,51,52,53]. While that baseline study [19] successfully demonstrated the feasibility of patient-level diagnostics, its scope was focused primarily on evaluating whether an explainable Vision Transformer framework could effectively aggregate slice-level features to yield anatomically plausible visual explanations.

The present study extends that foundational work conceptually rather than structurally. Instead of developing a new model architecture to re-verify if deep learning can classify rotator cuff tears, this study shifts the core research question to a more granular, engineering-focused inquiry: Which specific MRI sequences and metadata inputs should be fused, and under what architectural conditions does fusion yield a genuine diagnostic benefit? This question is highly relevant to translation because clinical musculoskeletal MRI protocols are inherently heterogeneous, consisting of multiple distinct acquisitions designed for separate diagnostic roles.

The exceptional performance of our single-sequence PDc ResNet baseline aligns perfectly with established orthopedic radiology practices, where coronal proton-density fat-suppressed series serve as the gold standard for evaluating supraspinatus tendon disruptions. The strong complementary performances of the T2 sagittal and PDt axial models further reflect their clinical utility in mapping fluid-tendon interfaces and transverse-plane cuff anatomy. Conversely, the finding that the full four-sequence model was outperformed by the C14M configuration suggests that T1-weighted coronal imaging may introduce weakly informative features or redundant noise for this specific binary classification endpoint. This finding must remain task-specific; T1-weighted imaging remains clinically indispensable for marrow characterization, muscle atrophy grading, surgical planning, and differential diagnosis.

### 4.3. Why Selected Sequence Sets May Outperform Full-Sequence Fusion

The empirical finding that the streamlined C14M model outperformed the complete, all-sequence protocol is compatible with several distinct machine learning and radiomic principles:

- Dimensionality and Parameter Pressure: Appending additional MRI sequences exponentially scales the input feature dimensionality. When utilizing independent backbone encoders per sequence, this increases trainable parameter pressure, escalating the risk of overfitting on a single-center cohort.
- Domain Heterogeneity: Distinct imaging planes and contrast weightings introduce substantial domain variance. Forcing a classifier to reconcile non-fat-suppressed anatomical structures (T1) with fluid-sensitive pathological markers (T2/PD) can introduce contradictory feature vectors that destabilize joint optimization space.
- Spurious Correlations: Small to mid-sized medical imaging datasets are inherently vulnerable to non-generalizable, site-specific artifacts. A sequence that is clinically informative for a human radiologist may still encode non-causal features that undermine deep learning stability for a narrow binary classification task.
- Diminishing Marginal Value: The utility of any single imaging modality is strictly bounded by what is already captured by the existing input set. A sequence may exhibit excellent performance when evaluated in isolation, yet become entirely redundant when combined with higher-capacity, complementary sequences.

This interpretation strongly echoes multi-sequence MRI representation learning trends observed outside the musculoskeletal domain. For example, Han et al. explicitly detailed the interference caused by cross-sequence redundancy and proposed structured sequence-ranking concepts to select optimal subsets for non-inferiority protocols [20]. Similarly, missing-modality brain tumor segmentation studies have routinely shown that the marginal utility of an imaging series is highly dynamic, depending entirely on the target anatomy, the specific task, and the fusion mechanics [24,25,26,27,28,29]. Rather than suggesting that certain sequences are clinically obsolete, these findings support the more cautious, evidence-based claim that deep learning input selection must be empirically tested rather than assumed a priori.

### 4.4. Metadata as Context Rather than a Standalone Signal

The poor diagnostic execution of the standalone metadata model (AUC=0.6104) demonstrates that basic clinical variables are wholly insufficient for direct rotator cuff diagnosis. However, injecting these exact same variables into the three-sequence C14 configuration yielded a substantial performance surge (ΔAUC=+0.0262; ΔF1-score=+0.0690). This dichotomy strongly supports a context-dependent interpretation of multimodal biomedical AI: structured demographic features do not act as independent predictors, but rather as a context-dependent signal whose measurable benefit, in this cohort, was confined to the headline three-sequence configuration rather than being a uniform property of metadata fusion; as shown by the paired confidence intervals in Section 3.5, most per-configuration metadata deltas were within cross-validation variability.

Equally informative are the negative performance deltas observed when metadata was appended to less optimal imaging combinations (e.g., the transitions from C13 to C13M and C05 to C05M). These performance drops demonstrate that metadata is not a universally beneficial attachment. When the underlying visual representation is suboptimal or poorly aligned with the target pathology, appending non-image variables can alter the model’s final decision boundary in an unfavorable direction.

This pattern mirrors broader insights from the image-EHR fusion literature, which frequently warns that non-image variables can inadvertently encode institutional workflows, shifting referral patterns, or chronological documentation biases rather than true causal physiological signals [32,33,34,35,36,37,38,39,40]. For instance, acquisition year or age may correlate with machine-specific protocols or degenerate age-related changes rather than acute tear dynamics. To test the specific concern that acquisition year could encode a temporal shortcut for the label, we cross-tabulated class against study year: tear prevalence was essentially identical in the two acquisition years (49.7% in 2024 versus 52.4% in 2025; $\chi^{2}\;=\;0.10,\;p\;=\;0.76;$ Fisher’s exact *p* = 0.86), indicating that study year carried no exploitable class signal in this cohort (see also Section 4.7). Consequently, external validation remains uniquely critical for metadata-enhanced models, as non-image clinical variables are notorious for generalizing less reliably across different healthcare networks than standardized imaging features.

### 4.5. Engineering Implications

From an engineering standpoint, our results validate the necessity of a staged, hierarchical evaluation pipeline for multimodal medical imaging research.


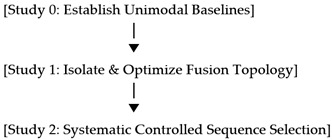


This structural decomposition minimizes the common flaw of blindly attributing performance variations to “multimodality” when the underlying cause may actually be driven by backbone capacity, dimensionality constraints, or volatile threshold-dependent metadata interactions.

Furthermore, our selection of late decision-level fusion for the final sequence-subset analysis carries distinct technical advantages. Late fusion enforces rigid architectural modularity: each sequence-specific encoder specializes in its own localized plane and contrast weighting before projecting an independent logit into a learned, weighted softmax combination ($l_{i}=\sum_{k}\alpha_{k}l_{i,k}$). This modularity shields the model from the catastrophic overfitting that often plagues high-dimensional concatenated feature spaces in data-constrained settings. However, because late fusion inherently discards early, fine-grained cross-sequence spatial correlations, this architectural choice represents a specialized empirical solution for our current cohort size, rather than a universal claim of superiority over early or intermediate fusion methods.

### 4.6. Clinical Implications

The optimized performance of the C14M configuration offers highly relevant insights for the design of efficient, sequence-aware computer-aided diagnosis (CADx) systems. Specifically, it demonstrates that a machine learning model optimized for binary rotator cuff tear classification does not require the entire multiplanar clinical imaging protocol to achieve peak internal diagnostic accuracy.

Critical Translation Distinction: Model input optimization and clinical protocol reduction are related but non-equivalent paradigms.

This finding must not be misconstrued as a medical recommendation to eliminate T1-weighted sequences from routine shoulder MRI protocols. Hospital imaging protocols are multi-purpose instruments designed to simultaneously rule out alternative pathologies, evaluate bone marrow edema, characterize muscle mass volumes, map labral integrity, and support complex pre-surgical planning—objectives that extend far beyond a binary tendon discontinuity task.

The actionable clinical implication is that developers of musculoskeletal AI architectures must explicitly report the exact sequence configurations utilized by their models and empirically justify their inputs. If subsequent external validations confirm that a streamlined T2 + PDc + PDt + metadata configuration maintains robust accuracy across diverse clinical centers and vendors, such a model could be safely deployed to drive high-throughput triage mechanisms or automated preliminary reporting pipelines, all while the full comprehensive protocol remains fully accessible to the reading radiologist for holistic interpretation.

### 4.7. Limitations

This study is bound by several important limitations that temper its immediate clinical generalizability:

- Single-Center Constraint: All evaluations were conducted on a single-center cohort of 199 patients without external validation. The near-perfect internal area under the curve metrics (AUC=0.9961 for C14M) must be interpreted with extreme caution, as medical imaging models routinely experience performance drops when exposed to external data. All results should therefore be read as model-development and internal-validation findings only. Moreover, because the tear/no-tear reference standard was itself derived from expert interpretation of the same MRI examinations that the network ingests (Section 2.1), these figures quantify concordance with expert clinical reads rather than accuracy against an independent, image-external gold standard such as arthroscopy or surgery.
- Binary Endpoint Simplification: A binary tear/no-tear target class fails to capture the true, highly nuanced clinical spectrum of rotator cuff pathology. Real-world management hinges on partial-thickness classification, tear measurements, tendon retraction distances, muscle atrophy scoring, and the long-term progression of fatty infiltration.
- Scanner Homogeneity: Although our fold partitioning eliminated slice-level data leakage, the pipeline did not test robustness across alternative scanner vendors, variations in magnetic field strength (1.5 T vs. 3.0 T), or heterogeneous institutional acquisition parameters. All examinations were in fact acquired on a single 1.5 T scanner from one vendor within a narrow acquisition window (78.9% of cases from 2024), so robustness to alternative vendors, field strengths and protocol generations remains entirely untested and is a primary target for external validation.
- Metadata Dependency: The structured clinical features were restricted to basic demographic variables available in the source dataset. Their diagnostic contributions may simply reflect localized demographic distributions or center-specific scheduling patterns, limiting their reproducibility elsewhere.
- Fusion-Family Boundary: The sequence-combination matrix in Study 2 was evaluated exclusively within the late decision-level fusion framework selected from Study 1. Alternative architectural families, hyper-scale backbones, self-supervised pretraining regimens, or multi-task optimization objectives might yield entirely different optimal sequence rankings. Consequently, the specific top ranking of C14M may be partly architecture-dependent rather than purely sequence-dependent, and the extent which it persists under intermediate or attention-based fusion could not be determined from the present experiments.
- Acquisition-Era Confounding: Acquisition year was included as a structed metadata feature and could, in principle, encode scanner-era or protocol effects rather than anatomy. Although the class-by-year cross-tabulation revealed no association between tear prevalence and study year in this cohort (Section 4.4), a formal ablation that removes the year feature and retrains the affected configurations was not performed here and is left to future work.
- Post hoc Model Selection: The headline configuration C14M was chosen as the best of 22 sequence/metadata configurations evaluated on the same outer folds used for reporting. Because this choice was made post hoc rather than inside the training folds, the between-configuration comparisons are hypothesis-generating and the corresponding differences are optimistic by construction; rigorous selection would require nested cross-validation, which we identify as future work.
- Qualitative Explainability: Visual heatmaps generated via Grad-CAM or transformer attention tracking remain strictly qualitative metrics. They serve as basic anatomical sanity checks but do not constitute mathematical proof of diagnostic reasoning, internal calibration, or causal inference. In particular, no quantitative localization metric (for example, intersection-over-union or a pointing-game score against radiologist-annotated regions of interest) was computed; the saliency maps therefore support anatomical plausibility only and must not be read as evidence of correct, lesion-level localization.

### 4.8. Future Work

To transition these findings toward trustworthy clinical translation, future research must prioritize validating the optimized C14M configuration across multi-institutional, heterogeneous external test sets that represent a wide array of scanner vendors and protocol variations. Additionally, subsequent modeling efforts should expand beyond binary classifications to target clinically richer, multi-task labels—such as differentiating partial-thickness from full-thickness tears, quantifying tendon retraction, and staging fatty infiltration. Transitioning to a multi-task learning paradigm may encourage deep encoders to construct more coherent, radiologically grounded representations rather than exploiting narrow binary shortcuts.

From an engineering perspective, future studies should evaluate alternative fusion paradigms, attention-driven multi-modal transformers, and missing-sequence robust architectures on significantly larger patient populations. Crucially, systematic sequence selection should be coupled with rigorous uncertainty estimation and model calibration assessments; a diagnostic model with an exceptional area under the curve can still prove clinically dangerous if its confidence metrics are poorly calibrated for downstream decision support. Finally, metadata fusion requires deep algorithmic bias and confounder analyses to ensure that clinical variables genuinely reinforce generalizable physiological markers rather than codifying institutional shortcuts. Several methodological extensions follow directly from the present analysis: (i) nested cross-validation, in which the sequence/metadata configuration is selected entirely within the training folds, to provide unbiased estimates of the selected model’s performance; (ii) a dedicated study-year ablation that removes the acquisition-year feature and retraining the affected configurations, confirming that metadata gains are anatomy rather than era-driven; (iii) a direct comparison of late fusion against intermediate and attention-based fusion families, to establish whether to optimal sequence subset is stable across architectures or partly architecture dependent; (iv) extension from the binary endpoint to clinically richer, multi-task labels (partial- versus full-thickness tears, tendon retraction, and fatty-infiltration staging); (v) multi-institutional external validation across scanner vendors and fields strengths; and (vi) quantitative explainability evaluation using intersection-over-union and pointing-game scores against radiologist-annotated regions of interests.

## 5. Conclusions

In this single-center shoulder MRI cohort, a hierarchical evaluation of single-sequence baselines, multimodal fusion topologies, and sequence-subset configurations identified a tailored three-sequence protocol—consisting of T2 TSE fat-suppressed sagittal, PDc TSE fat-suppressed coronal, and PDt TSE fat-suppressed axial/transverse imaging—coupled with structured clinical metadata (C14M) as the most robust input configuration for binary rotator cuff tear classification. Crucially, the complete four-sequence metadata-enhanced protocol failed to outperform this optimized subset.

These empirical findings demonstrate that a targeted, sequence-aware multimodal fusion approach can be more informative and stable than the indiscriminate aggregation of an entire clinical imaging protocol. Furthermore, the diagnostic utility of clinical metadata was highly combination-dependent, yielding its maximum performance gain only when paired with the fluid-sensitive and proton-density-informed sequence framework (T2+PDc+PDt). Ultimately, extensive multi-institutional external validation remains imperative to verify the generalizability of these optimized feature spaces before clinical deployment or the consideration of diagnostic protocol modifications.

## Figures and Tables

**Table 1:** Baseline demographic characteristics and structured clinical metadata of the study cohort (n=199). Continuous variables are expressed as mean ± standard deviation (SD).

Characteristic	Value (*n* = 199)
Diagnosis, *n* (%)	
- Rotator Cuff Tear	100 (50.3%)
- No Tear (Control)	99 (49.7%)
Sex, *n* (%)	
- Female	118 (59.3%)
- Male	81 (40.7%)
Age (years), mean ± SD	50.2 ± 18.1
BMI (kg/m^2^), mean ± SD	26.5 ± 3.2
Laterality, *n* (%)	
- Right	74 (37.2%)
- Left	56 (28.1%)
- Unspecified	69 (34.7%)
Study Year, *n* (%)	
- 2024	157 (78.9%)
- 2025	42 (21.1%)

**Table 2:** Technical acquisition parameters and scanner specifications across the four evaluated shoulder MRI sequences. TR: repetition time, TE: echo time, TSE: turbo spin-echo.

Sequence	Sequence Description	TR (ms)	TE (ms)	Slice Thickness (mm)	Matrix Size
T1	T1 TSE Coronal	669.2	20.1	4.3	256 × 256
T2	T2 TSE Fat-Suppressed Sagittal	4258.2	71.0	4.3	256 × 256
PDc	PD TSE Fat-Suppressed Coronal	3115.7	34.1	4.3	256 × 256
PDt	PD TSE Fat-Suppressed Transverse (Axial)	3137.3	26.0	4.3	256 × 256

**Table 3:** Methodological outline of the hierarchical experimental framework, detailing the specific objectives, input modalities, and primary analytical outcomes for Studies 0, 1, and 2.

Study Phase	Purpose/Objective	Input Modalities	Primary Outcome/Key Finding
Study 0	Baseline unimodal evaluation and ResNet50 vs. ViT architecture comparison	Individual MRI sequences (T1, T2, PDc, PDt) and a standalone clinical metadata model	PDc ResNet50 identified as the optimal single-sequence baseline
Study 1	Benchmarking of alternative multimodal fusion topologies	Full clinical protocol (all four MRI sequences combined with clinical metadata)	ResNet50 late decision fusion selected based on superior global AUC performance
Study 2	Systematic sequence-subset optimization and metadata impact analysis	Pairwise, three-sequence, and full-protocol MRI combinations (with and without metadata)	C14M configuration (T2 + PDc + PDt + metadata) established as the peak-performing diagnostic model

**Table 4:** Diagnostic classification performance of unimodal baseline models in Study 0 across individual MRI sequences and standalone clinical metadata. CI: confidence interval, MCC: Matthews correlation coefficient, R: ResNet50, V: Vision Transformer.

Model	Input Modality	AUC	Accuracy	Sensitivity	Specificity	F1-Score	MCC	95% CI for AUC
**S0-R-PDc**	**PDc**	**0.9834**	**0.9498**	**0.9700**	0.9293	**0.9515**	**0.9028**	0.9508–0.9930
S0-R-T2	T2	0.9780	0.9096	0.9192	0.8990	0.9110	0.8271	0.9281–0.9825
S0-R-PDt	PDt	0.9742	0.9095	0.8794	**0.9394**	0.9064	0.8221	0.9400–0.9890
S0-V-PDt	PDt	0.9663	0.9092	0.9293	0.8889	0.9106	0.8195	0.9410–0.9878
S0-V-T2	T2	0.9594	0.9396	0.9394	**0.9394**	0.9388	0.8815	0.9337–0.9859
S0-V-PDc	PDc	0.9564	0.8993	0.9094	0.8889	0.9002	0.7991	0.9104–0.9775
S0-R-T1	T1	0.9543	0.8743	0.8604	0.8889	0.8755	0.7491	0.8958–0.9698
S0-V-T1	T1	0.9069	0.8341	0.8515	0.8182	0.8372	0.6686	0.8399–0.9357
S0-Meta	Metadata	0.6104	0.5430	0.8791	0.2020	0.6575	0.0738	0.4855–0.6477

**Table 5:** Comparative diagnostic performance metrics of alternative multimodal fusion topologies in Study 1 utilizing the complete four-sequence MRI protocol combined with clinical metadata.

Strategy	AUC	Accuracy	Sensitivity	Specificity	Precision	F1-Score	MCC	95% CI for AUC
**ResNet Late Decision Fusion**	**0.9909**	0.9346	0.9495	0.9192	0.9235	0.9355	0.8715	0.9699–0.9974
ResNet Feature Concatenation	0.9753	**0.9498**	0.9495	**0.9495**	**0.9545**	**0.9502**	**0.9030**	0.9416–0.9920
ViT Late Decision Fusion	0.9722	0.9196	**0.9697**	0.8687	0.8828	0.9237	0.8444	0.9467–0.9914
ResNet Attention-Weighted Fusion	0.9694	0.9095	0.9498	0.8687	0.8867	0.9138	0.8282	0.9309–0.9840
ViT Feature Concatenation	0.9614	0.8898	0.9599	0.8182	0.8520	0.8996	0.7928	0.9118–0.9755
ResNet Early Two-Sequence Fusion	0.9164	0.8136	0.7986	0.8283	0.8244	0.8095	0.6300	0.8231–1.0000
ResNet Compact Bilinear Pooling	0.9056	0.8543	0.9195	0.7879	0.8221	0.8635	0.7234	0.8297–0.9815
ViT Token-Level Early Fusion	0.5436	0.5025	1.0000	0.0000	0.5052	0.6689	0.0000	0.4248–0.6624

**Table 6:** Combinatorial performance matrix in Study 2 evaluating all possible pairwise, three-sequence, and full-protocol MRI subsets with (“M”) and without clinical metadata. Models are sorted by descending Area Under the ROC Curve (AUC).

Model ID	Sequence Combination	Clinical Metadata	AUC	Accuracy	Sensitivity	Specificity	Precision	F1-Score	MCC
C14M *	**T2 + PDc + PDt**	**Yes**	**0.9961**	**0.9600**	**0.9798**	0.9394	0.9475	**0.9618**	**0.9238**
C11M	T1 + T2 + PDc	Yes	0.9918	0.9346	0.9394	0.9293	0.9333	0.9350	0.8720
C05	T1 + T2	No	0.9917	0.9546	0.9697	0.9394	0.9423	0.9557	0.9099
C15M	T1 + T2 + PDc + PDt	Yes	0.9909	0.9346	0.9495	0.9192	0.9235	0.9355	0.8715
C13	T1 + PDc + PDt	No	0.9905	0.9195	0.8895	0.9495	0.9518	0.9148	0.8473
C08M	T2 + PDc	Yes	0.9899	0.9345	0.9394	0.9293	0.9335	0.9332	0.8748
C10M	PDc + PDt	Yes	0.9885	0.9295	0.9596	0.8990	0.9071	0.9317	0.8624
C12M	T1 + T2 + PDt	Yes	0.9866	0.9597	0.9697	0.9495	0.9506	0.9597	0.9202
C08	T2 + PDc	No	0.9866	0.9447	0.9697	0.9192	0.9236	0.9458	0.8911
C11	T1 + T2 + PDc	No	0.9864	0.9146	0.9697	0.8586	0.8751	0.9196	0.8352
C15	T1 + T2 + PDc + PDt	No	0.9863	0.9395	0.9192	**0.9596**	**0.9592**	0.9374	0.8822
C06M	T1 + PDc	Yes	0.9834	0.8943	0.9498	0.8384	0.8695	0.9024	0.8044
C09M	T2 + PDt	Yes	0.9833	0.9246	0.9394	0.9091	0.9168	0.9258	0.8536
C13M	T1 + PDc + PDt	Yes	0.9824	0.9044	0.9091	0.8990	0.9074	0.9022	0.8188
C10	PDc + PDt	No	0.9817	0.9195	0.9293	0.9091	0.9160	0.9190	0.8453
C12	T1 + T2 + PDt	No	0.9806	0.8996	0.9798	0.8182	0.8481	0.9083	0.8113
C07M	T1 + PDt	Yes	0.9796	0.9094	0.9293	0.8889	0.8942	0.9108	0.8208
C06	T1 + PDc	No	0.9775	0.9045	0.9302	0.8788	0.8881	0.9069	0.8137
C07	T1 + PDt	No	0.9768	0.8994	0.9002	0.8990	0.9111	0.9010	0.8076
C09	T2 + PDt	No	0.9741	0.8996	0.8898	0.9091	0.9107	0.8994	0.8006
C05M	T1 + T2	Yes	0.9731	0.9247	0.9798	0.8687	0.8844	0.9292	0.8555
C14	T2 + PDc + PDt	No	0.9699	0.8943	0.8889	0.8990	0.9028	0.8928	0.7942

* Denotes that C14M was the top-ranked configuration among all 22 evaluated sequence/metadata combinations. Because this configuration was identified post hoc on the same outer folds used for evaluation, between-configuration differences are reported descriptively rather than as confirmatory significance tests, owing to post-selection (winner’s-curse) bias. On the pooled out-of-fold predictions, paired DeLong comparisons did not reach statistical significance against either the full four-sequence protocol C15M (ΔAUC = 0.0056; z = 1.26; *p* = 0.21) or the single sequence PDc baseline (ΔAUC = 0.0176; z = 1.60; *p* = 0.11); these descriptive p-values should be interpreted with caution given the post hoc selection.

**Table 7:** Out-of-fold diagnostic performance breakdown across the three-fold stratified outer cross-validation splits for the top-performing C14M configuration (T2+PDc+PDt+Metadata).

Fold	AUC	Accuracy	Sensitivity	Specificity	Precision	F1-Score	NPV	MCC
Fold 0	0.9929	0.9254	1.0000	0.8485	0.8718	0.9315	1.0000	0.8601
Fold 1	0.9991	0.9848	1.0000	0.9697	0.9706	0.9851	1.0000	0.9701
Fold 2	0.9963	0.9697	0.9394	1.0000	1.0000	0.9688	0.9429	0.9411
**Mean**	**0.9961**	**0.9600**	**0.9798**	**0.9394**	**0.9475**	**0.9618**	**0.9810**	**0.9238**

**Table 8:** Isolated marginal performance deltas (Δ) demonstrating the exact positive and negative diagnostic impacts of integrating structured clinical metadata across precisely matched MRI sequence combinations.

Comparison Pair	Sequence Combination	ΔAUC	ΔF1-Score	ΔPrecision	ΔSensitivity
**C14 → C14M**	**T2 + PDc + PDt**	**+0.0262**	**+0.0690**	+0.0447	**+0.0909**
C09 → C09M	T2 + PDt	+0.0093	+0.0264	+0.0061	+0.0496
C10 → C10M	PDc + PDt	+0.0067	+0.0127	−0.0089	+0.0303
C12 → C12M	T1 + T2 + PDt	+0.0060	+0.0515	**+0.1025**	−0.0101
C06 → C06M	T1 + PDc	+0.0058	−0.0045	−0.0185	+0.0196
C11 → C11M	T1 + T2 + PDc	+0.0055	+0.0154	+0.0582	−0.0303
C15 → C15M	T1 + T2 + PDc + PDt	+0.0046	−0.0019	−0.0357	+0.0303
C08 → C08M	T2 + PDc	+0.0033	−0.0126	+0.0099	−0.0303
C07 → C07M	T1 + PDt	+0.0028	+0.0098	−0.0169	+0.0291
C13 → C13M	T1 + PDc + PDt	−0.0081	−0.0127	−0.0444	+0.0196
C05 → C05M	T1 + T2	−0.0187	−0.0265	−0.0579	+0.0101

Paired (fold-matched) bootstrap 95% confidence intervals for ΔAUC (2000 resamples; fixed seed; resampling performed within each cross-validation fold), reported here as [lower, upper]: C14 → C14M [+0.0158, +0.0640]; C09 → C09M [+0.0028, +0.0486]; C10 → C10M [+0.0030, +0.0217]; C12 → C12M [−0.0048, +0.0192]; C06 → C06M [−0.0165, +0.0189]; C11 → C11M [−0.0015, +0.0133]; C15 → C15M [−0.0057, +0.0122]; C08 → C08M [−0.0136, +0.0040]; C07 → C07M [−0.0075, +0.0381]; C13 → C13M [−0.0309, −0.0021]; C05 → C05M [−0.0382, −0.0067]. Only five intervals exclude zero: C14 → C14M, C09 → C09M and C10 → C10M (positive) and C05 → C05M and C13 → C13M (negative); the remaining six intervals span zero and the corresponding deltas are therefore withing cross-validation sampling variability.

## Data Availability

The data presented in this study are available on request from the corresponding author.
